# The Clinical Outcomes Among Patients Under 60 Years Old with Lynch Syndrome: Variations Based on Different Mutation Patterns

**DOI:** 10.3390/ijms26073383

**Published:** 2025-04-04

**Authors:** Calin Muntean, Vasile Gaborean, Razvan Constantin Vonica, Alaviana Monique Faur, Vladut Iosif Rus, Ionut Flaviu Faur, Catalin Vladut Ionut Feier

**Affiliations:** 1 Medical Informatics and Biostatistics, Department III-Functional Sciences, “Victor Babes” University of Medicine and Pharmacy Timisoara, Eftimie Murgu Square No. 2, 300041 Timisoara, Romania; cmuntean@umft.ro; 2 Thoracic Surgery Research Center, “Victor Babeş” University of Medicine and Pharmacy Timişoara, Eftimie Murgu Square No. 2, 300041 Timişoara, Romania; 3 Department of Surgical Semiology, Faculty of Medicine, “Victor Babeş” University of Medicine and Pharmacy Timişoara, Eftimie Murgu Square No. 2, 300041 Timişoara, Romania; 4 Preclinical Department, Faculty of Medicine, “Lucian Blaga” University of Sibiu, 550169 Sibiu, Romania; razvanconstantin.vonica@ulbsibiu.ro; 5 Department of Oncology, Elysee Hospital, 510040 Alba Iulia, Romania; 6 Faculty of Medicine, “Victor Babeş” University of Medicine and Pharmacy Timişoara, 300041 Timişoara, Romania; alaviana.faur@student.umft.ro (A.M.F.); iosif.rus@student.umft.ro (V.I.R.); 7 IInd Surgery Clinic, Timisoara Emergency County Hospital, 300723 Timisoara, Romania; flaviu.faur@umft.ro; 8 Department X of General Surgery, “Victor Babes” University of Medicine and Pharmacy Timisoara, 300041 Timisoara, Romania; 9 Abdominal Surgery and Phlebology Research Center, “Victor Babes” University of Medicine and Pharmacy, 300041 Timisoara, Romania; catalin.feier@umft.ro; 10 First Surgery Clinic, “Pius Brinzeu” Clinical Emergency Hospital, 300723 Timisoara, Romania

**Keywords:** Lynch syndrome, mismatch repair genes, hereditary colorectal cancer, microsatellite instability, young-onset CRC

## Abstract

Lynch syndrome (LS)—also known as Hereditary Non-Polyposis Colorectal Cancer (HNPCC)—is caused by pathogenic germline mutations in DNA mismatch repair (MMR) genes such as *MLH1*, *MSH2*, *MSH6*, and *PMS2*. Although it accounts for only 1–5% of all colorectal cancers (CRCs), LS presents with a particularly high lifetime cancer risk and often occurs at younger ages. Identifying LS in patients under 60 years old is crucial for targeted surveillance and early interventions. Variations in clinical presentation and prognosis may exist based on the specific gene mutated, yet these patterns are not fully elucidated. This review aims to synthesize data on clinical outcomes among LS patients under 60, with an emphasis on how different MMR gene mutation patterns might influence prognosis, survival, and treatment decisions. Five population-based studies examining CRC patients younger than 60 years were included according to predefined eligibility criteria. Two independent reviewers screened and extracted data focusing on MMR deficiency detection methods (microsatellite instability [MSI] and/or immunohistochemistry [IHC]), rates of confirmed germline mutations, frequency of BRAF testing, and clinical endpoints such as stage distribution, survival outcomes, and recurrence. Risk of bias was assessed using standardized tools appropriate to each study design. The synthesis focused on comparing outcomes among individuals with *MLH1*, *MSH2*, *MSH6*, and *PMS2* mutations, as well as delineating the proportion of patients with sporadic MSI under 60 years of age. Across the five studies, MSI positivity in CRC patients under 60 years ranged from 7.5% to 13%. The frequency of confirmed germline MMR mutations varied between 0.8% and 5.2% in specific cohorts, aligning with LS prevalence estimates of 1–5%. Different mutation patterns correlated with some variation in clinical presentation. Cases with *MSH2* and *MLH1* mutations more frequently exhibited synchronous or metachronous tumors, while *MSH6* and *PMS2* mutations displayed more heterogeneous IHC patterns. Where survival data were provided, LS patients under 60 years had better overall survival compared to MMR-proficient individuals, though some studies also noted a potential lack of benefit from standard 5-fluorouracil adjuvant therapy in MMR-deficient tumors. Screening by MSI or by IHC—supplemented with BRAF mutation testing to exclude sporadic MSI—facilitates early detection of LS in CRC patients under 60 and highlights notable differences between mutation types. Although overall outcomes for LS patients can be favorable, especially for stage II disease, the precise impact of each specific mutated gene on clinical course remains heterogeneous. Future large-scale prospective studies are needed to clarify optimal screening protocols and individualized treatment strategies for LS patients under 60.

## 1. Introduction

Lynch syndrome (LS), characterized by germline mutations in DNA mismatch repair genes, leads to a significantly increased risk of developing various forms of cancer [1]. This genetic disorder is associated with a hereditary predisposition to cancer development, primarily colorectal and endometrial cancers. The pathogenesis of LS involves the accumulation of mutations due to the failure of the mismatch repair system, which normally corrects errors during DNA replication. LS, also designated Hereditary Nonpolyposis Colorectal Cancer (HNPCC), arises from germline pathogenic variants in the DNA mismatch repair (MMR) genes—most frequently in MLH1 or MSH2, but also MSH6 and PMS2 [1,2]. Individuals with LS are prone to earlier onset colorectal cancer (CRC), often before the age of 60, and exhibit a heightened lifetime risk of multiple malignancies, including endometrial, gastric, ovarian, and ureteric tumors [3,4]. Historically, family history-based criteria, such as the Amsterdam I/II criteria and the Revised Bethesda guidelines, have aided clinical recognition of LS, yet up to half of the mutation carriers remain undetected in routine practice [5]. In response, many institutions now adopt universal or near-universal screening for MMR deficiency via microsatellite instability (MSI) or immunohistochemistry (IHC) in newly diagnosed CRC, especially among patients under 60 [6,7]. Still, despite broad agreement on the importance of tumor-based screening, debate persists regarding optimal thresholds (e.g., universal testing versus selective strategies) and the most efficient testing modalities—MSI, IHC, or combined approaches [8,9].

In younger CRC cohorts, the prevalence of MMR-deficient tumors has been reported to range between 7% and 13%, a rate substantially higher than in older populations [10,11]. Many of these MMR-deficient tumors are ultimately classified as sporadic if they harbor BRAF c.1799T>A (p.V600E) mutations or show hypermethylation of the MLH1 promoter [12]. Nevertheless, among the remaining “red flag” cases—those lacking BRAF mutations or MLH1 methylation—a considerable number prove to carry germline MMR gene mutations indicative of LS [13]. Several large-scale implementations of universal or near-universal tumor testing have demonstrated improved detection of Lynch syndrome at a younger age, translating into earlier interventions for at-risk relatives [14,15]. Encouragingly, cost-effectiveness analyses suggest that routine screening of CRC patients under 60 is justifiable based on averted future cancer treatments and the extended life-years gained through prophylactic measures [16].

In terms of clinical outcomes, multiple retrospective and prospective series have observed that MMR-deficient CRC—whether sporadic or Lynch-related—tends to confer a better prognosis than MMR-proficient disease, particularly for localized Stage II cancers [17,18]. Part of this survival advantage might stem from an active immune response triggered by the high neoantigen load in MMR-deficient tumors [11,19]. Consequently, MMR deficiency may inform both prognosis and treatment selection, as growing evidence suggests a limited or unclear benefit from conventional 5-fluorouracil (5-FU)-based chemotherapy in these tumors [10,20]. For patients under 60, accurate detection of MMR deficiency may therefore not only confirm LS but also influence postoperative management strategies, such as more targeted immunotherapy approaches for advanced cases [11].

From a molecular standpoint, MLH1 or MSH2 mutations historically account for most Lynch syndrome diagnoses, yet MSH6 and PMS2 have gained increasing recognition for their contributions to LS cases displaying subtle or atypical IHC patterns [2,13]. For example, partial or heterogeneous staining, particularly for MSH6 or PMS2, can lead to false-negative interpretations unless pathologists carefully examine multiple tumor regions [15]. While universal screening protocols are increasingly endorsed, real-world implementation varies by region, with some centers opting for selective testing (e.g., patients under 60 or with a strong family history).

Young CRC patients with MLH1 or MSH2 mutations often present with a stronger family history of early-onset cancers and can develop synchronous or metachronous lesions. Meanwhile, MSH6 or PMS2 mutation carriers—though numerically fewer—may manifest at slightly older ages or with more ambiguous IHC findings [8,14]. Despite the growing literature, questions remain about whether certain gene-specific subgroups experience more frequent extracolonic malignancies or respond differently to novel therapeutics (e.g., checkpoint inhibitors) [11,19,20,21,22]. Against this backdrop, this review, focusing on individuals under 60, aims to describe how MMR mutations shape clinical trajectories, to highlight best practices in screening, and to suggest areas for future research aimed at refining LS management.

## 2. Results

Table 1 summarizes the key features of the five included studies, highlighting their designs, geographic settings, sample sizes, and methods of detecting mismatch repair (MMR) deficiency. All the studies targeted CRC patients under 60 years of age or included sufficient data on younger patients to permit extraction of relevant outcomes. Notably, four out of five were conducted in Western Australia or Victoria, reflecting a robust interest within Australia in screening for Lynch syndrome (LS).

Each study used a combination of molecular and immunohistochemical assays to identify MMR defects. Schofield et al. [21,22,23,24] and Watson et al. [25] predominantly relied on single-marker or pentaplex MSI panels, supplemented by *BRAF V600E* mutation testing to exclude sporadic MSI. They subsequently performed IHC for the four major MMR proteins (*MLH1*, *PMS2*, *MSH2*, *MSH6*) in MSI-positive, *BRAF*-negative (“red flag”) tumors to guide germline testing referral. In contrast, Ward et al. [24] and Buchanan et al. [22] integrated multi-locus MSI panels (such as the Bethesda panel) and IHC from the outset. Each study reported a slightly different threshold for diagnosing MSI-high; for example, Schofield’s 2009–2010 cohort used MSI positivity at ≥2 mononucleotide markers, while the earliest Schofield paper tested solely *BAT-26* for efficiency.

Table 2 focuses on the proportion of under-60 CRC patients who tested positive for microsatellite instability (MSI+), the fraction that were *BRAF*-positive (and thus likely sporadic), and the yield of confirmed germline mutations. While definitions of MSI and number of markers varied among studies, the reported MSI+ rates consistently fall within a 7–13% range for younger CRC patients. An outlier is Schofield et al. [23], which found 26% MSI positivity in a smaller prospective cohort; the authors ascribed this higher rate partly to selective referral patterns and the inclusion of high-suspicion tumors. In that same cohort, 35.7% of MSI+ tumors harbored a *BRAF* mutation, consistent with a sporadic pathway via *MLH1* hypermethylation.

**Figure 1:**
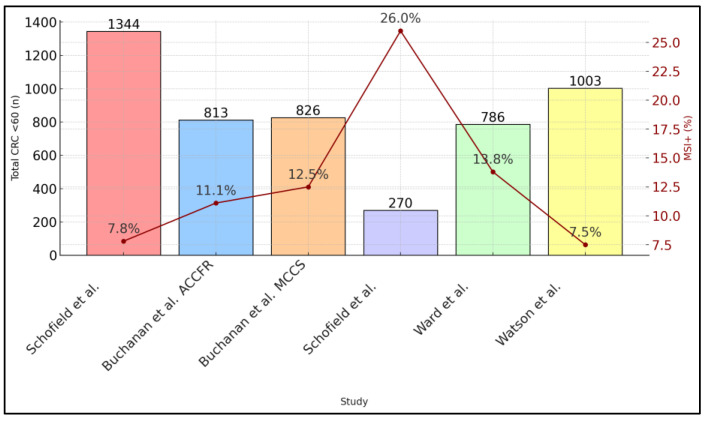
MSI+ percentage and total CRC under 60 [21,22,23,24,25].

After excluding *BRAF*-positive cases, each study directed its “red flag” tumors—MSI+ and *BRAF* wild-type—for germline MMR gene testing (if consent was obtained). Schofield et al. [21] identified a total of 36 carriers among 98 red-flag tumors, whereas the second Schofield program [23] found 15 carriers among 31 tested. Although Ward et al. [24] used a slightly different methodology (IHC first), they also identified a ∼0.8% LS prevalence. Watson et al. [25] reported that 18 patients in their cohort were already known carriers; an additional 3 were newly identified, but not all tumors were tested comprehensively, as presented in Figure 1.

In Table 3, we outline how each study characterized the distribution of pathogenic mutations within the four primary mismatch repair (MMR) genes—*MLH1*, *MSH2*, *MSH6*, and *PMS2*—and the corresponding immunohistochemistry (IHC) patterns. Across all five papers, *MLH1* and *MSH2* emerged as the most commonly mutated genes, consistent with classical Lynch syndrome prevalence. However, each study reported at least a few carriers of *MSH6* and *PMS2*, underscoring the importance of screening all four proteins in the IHC panel.

Conventional IHC teaching is that *MLH1* loss is typically accompanied by loss of PMS2 expression, while *MSH2* loss correlates with MSH6 deficiency. This holds true in most of the “red flag” tumors across the studies. Yet, several publications (notably Watson et al. and Schofield et al. [21]) highlighted atypical patterns. For instance, Schofield et al. [23] reported multiple cases of single-protein loss in PMS2 or MSH6, especially in younger patients who ultimately tested positive for germline mutations. Watson et al. [25] detailed heterogenous or “clonal” staining patterns—particularly for MSH6—sometimes leading to initial misinterpretation of “normal” expression. This underscores the complexity of IHC interpretation and the need for repeated or confirmatory testing. Of note, Ward et al. [24] primarily assessed only *MLH1* and *MSH2* with IHC, missing potential single-protein defects in MSH6 or PMS2.

Table 4 compiles the key clinical outcomes in younger CRC patients with MMR-deficient versus MMR-proficient tumors, as reported in the included studies. Although exact staging distributions and survival metrics vary, a consistent theme emerges: MMR-deficient patients under 60 typically present with fewer metastatic tumors (Stage IV) and demonstrate better overall survival. Schofield et al. [21] revealed that, while most MSI+ patients were indeed Stage II or III at diagnosis, their overall prognosis tended to be more favorable, corroborating prior evidence that MMR deficiency often confers a better outcome. Ward et al. [24] used multivariate modeling to confirm MMR deficiency as an independent positive prognostic factor, even after adjusting for stage and vascular invasion.

Buchanan et al. [22] further noted that these survival benefits can persist into the 5-year follow-up period, although subgroup analyses hinted that the advantage might be most pronounced for node-negative (Stage II) disease. Interestingly, multiple authors observed minimal incremental benefit from 5-fluorouracil (5-FU)-based chemotherapy among MMR-deficient patients, raising the possibility that standard adjuvant regimens could be de-escalated or re-evaluated in these subpopulations. Meanwhile, Watson et al. [25] highlighted that LS carriers (versus sporadic MSI) might have even stronger survival benefits, though firm conclusions were tempered by small sample sizes. Notably, the right-sided location (proximal colon) was common across MMR-deficient subsets, which is consistent with the recognized clinicopathological features of Lynch syndrome.

## 3. Discussion

### 3.1. Summary of Evidence

The novelty of this review lies in its targeted examination of clinical outcomes specifically among Lynch syndrome patients under the age of 60, emphasizing how different mutation patterns in the MMR genes influence prognosis, survival, and treatment decisions. This focus is particularly innovative as it addresses a gap in the existing literature by dissecting the outcomes based on distinct genetic profiles within a younger cohort, offering potentially new insights into personalized medical approaches and earlier intervention strategies for this high-risk group.

The primary finding across the included studies is that an appreciable fraction (ranging from ~7% to 13%) of young CRC patients exhibit MSI-High or loss of MMR protein expression. Furthermore, after excluding likely sporadic cases via *BRAF* or hypermethylation analysis, a significant subset of these individuals harbors a germline mutation consistent with Lynch syndrome—confirming that younger age of onset is a strong predictor of hereditary disease. Variations in reported LS prevalence partly reflect differences in screening thresholds, geographic catchment, and whether universal or selective testing was adopted. Nonetheless, each study underscores the importance of rigorous screening protocols to identify this at-risk population.

A second key theme is the correlation between MMR deficiency and better clinical outcomes. Across diverse cohorts, MMR-deficient patients, including confirmed LS carriers, showed notably improved survival relative to MMR-proficient counterparts—especially in localized (Stage II) disease. This survival advantage aligns with the prior literature indicating that the robust immune response elicited by MMR-deficient tumors may account for a more indolent course. However, the magnitude of benefit and precise biological mechanisms remain subjects of ongoing investigation. Of particular clinical interest is the possibility that standard 5-FU–based chemotherapy regimens do not confer the same benefit in these patients, a finding that, if confirmed by prospective trials, may suggest de-escalation of cytotoxic therapy for certain LS patients.

With respect to variations in mutation patterns, the majority of inherited cases continue to involve *MLH1* or *MSH2* genes; however, *MSH6* and *PMS2* deficiencies do occur and can be underestimated by incomplete or misinterpreted immunohistochemistry. Multiple authors noted that MSH6 or PMS2 protein losses can be focal or patchy, leading to potential misclassification unless slides are carefully reviewed. Moreover, these latter genes sometimes associate with a milder clinical phenotype or slightly later onset. The included studies collectively emphasize the value of using a four-antibody IHC panel (*MLH1*, *MSH2*, *MSH6*, *PMS2*) rather than limiting testing to the historically more common *MLH1* and *MSH2* proteins. Such thoroughness is especially relevant for ensuring no LS carriers are overlooked.

Despite valuable insights, the studies have inherent methodological differences. For instance, some used single-marker MSI testing (e.g., *BAT-26*) while others used multi-marker panels. Additionally, some were retrospective, whereas others adopted prospective or state-wide screening systems. These variations limit direct comparisons and highlight a need for standardized, protocol-driven research to define best practices for diagnosing LS among younger CRC patients. Nevertheless, the convergent message is clear: universal or near-universal tumor testing in patients under 60 significantly improves LS detection, and mutation-specific differences in outcomes warrant further investigation. This approach can facilitate evidence-based decisions about genetic testing, surveillance for extracolonic malignancies, and personalized adjuvant therapy strategies.

In a detailed analysis of colorectal cancer patients, the study by Matthew B Yurgelun et al. [26] highlighted the significant presence of germline cancer susceptibility gene mutations, with 9.9% of CRC patients carrying pathogenic mutations, including a 3.1% prevalence of Lynch syndrome. They discovered that among the LS-associated colorectal cancers, a striking 96.6% showed abnormal microsatellite instability or mismatch repair (MMR) deficiency results. Conversely, 7.0% of patients harbored non-LS gene mutations, underscoring a complex genetic landscape where 2.2% carried mutations in high-penetrance genes such as APC and BRCA1/2 without prior suggestive clinical histories. This suggests a broader spectrum of genetic predispositions in CRC beyond LS, necessitating comprehensive genetic testing regardless of clinical indicators. In a similar manner, the study by Valentin Zumstein et al. [27] at a single center emphasized the efficacy of immunohistochemical screening to detect Lynch syndrome, revealing that 15.0% of CRC tumors demonstrated loss of MMR protein expression and 6.0% of CRCs were potential Lynch syndrome cases. This high detection rate, coupled with findings that 40% of the genetically tested potential Lynch syndrome carriers were diagnosed for the first time, advocates for the importance of routine MMR status evaluation in CRC cases. The study further supports the need for protocols that integrate MMR testing and genetic counseling as essential components of CRC management to identify individuals at increased risk efficiently.

In a retrospective cohort study by Dan Li et al. [28], the effectiveness of universal versus age-restricted screening for Lynch syndrome using mismatch repair immunohistochemistry (MMR IHC) was assessed among 3891 CRC patients. The findings revealed that the diagnostic yield of LS decreased significantly with age, identifying only 1.6% of LS cases after age 80. The study highlighted that using age 75 as the upper limit for screening could miss about 4.8% of LS cases but would reduce the screening need by 27.1% of the cases. This suggests that tailoring the upper age limit for LS screening might be practical to optimize resource use without significantly compromising diagnostic yield.

In a similar manner, the study by Heather Hampel et al. [13] evaluated the efficiency of up-front tumor sequencing (TS) as a potential replacement for the current sequential testing approach for LS screening in CRC. The research demonstrated that TS had a superior sensitivity of 100% compared to 89.7% for the combined MSI testing and IHC staining followed by BRAF mutation analysis. Importantly, TS also identified 284 cases with mutations relevant for stage IV CRC treatment, showing its additional benefit in therapeutic decision-making. Both studies underscore the evolving landscape of genetic screening for LS in CRC, with the move towards more efficient and potentially cost-effective methodologies that could streamline the diagnostic process and enhance patient care.

In a population-based study by Noora Porkka et al. [29], Lynch-like syndrome (LLS) was characterized through a detailed molecular and clinical analysis of colorectal carcinomas in Central Finland. The study identified 14 cases of LLS among 107 MMR-deficient tumors, representing 13% of the cohort. These tumors, which lacked MLH1 promoter methylation and were not associated with germline mutations, presented distinct features such as a mean diagnosis age of 65 years and a high frequency of CpG Island Methylator Phenotype (CIMP), with 13 out of 14 being CIMP-positive. Additionally, these LLS tumors demonstrated a notable absence of the BRAF V600E mutation, setting them apart from other MLH1-methylated CRCs. This distinct molecular profile underscores the complexity of diagnosing and managing LLS compared to Lynch syndrome, where tumors generally present with specific germline mutations. In a similar manner, the study by Venetia R Sarode et al. [30] explored the diagnostic challenges of using IHC for screening Lynch syndrome in a clinical setting, emphasizing the significance of indeterminate IHC results. They found that 50% of the cases with indeterminate expression were later confirmed to have Lynch syndrome through germline mutation studies. This high proportion of LS detection among indeterminate IHC results underscores the necessity for rigorous follow-up in cases where IHC does not conclusively diagnose LS.

Moreover, recent research and regulatory updates in 2023–2024 have significantly advanced LS screening and the use of immunotherapy for LS-associated cancers. Studies suggest that immunotherapy, particularly checkpoint inhibitors, could prevent cancer in individuals at increased genetic risk, prompting plans for further clinical trials [31]. Concurrently, the FDA’s approval of pembrolizumab for MSI-H and dMMR tumors represents a pivotal development, extending treatment options for LS-related cancers [32]. Additionally, NICE guidelines have refined diagnostic approaches for LS in endometrial cancer patients, emphasizing immunohistochemical testing and genetic confirmation, which could enhance early detection and treatment [33]. These advancements reflect a growing integration of genetic insights into clinical practice, aiming to improve outcomes for patients with Lynch syndrome.

The study underscores the clinical utility of LS screening in young CRC patients, highlighting how early detection can significantly influence patient management and outcomes in general surgery. By utilizing methods such as MSI and IHC, supplemented with BRAF testing, surgeons and oncologists can identify LS in patients under 60, facilitating targeted surveillance and potentially lifesaving interventions. The findings suggest that different genetic mutations within the MMR genes—MLH1, MSH2, MSH6, and PMS2—correlate with variations in clinical presentation, including the frequency of synchronous or metachronous tumors and survival outcomes. Such detailed understanding allows for more tailored surgical and adjuvant treatment strategies, particularly considering the observed variability in response to standard chemotherapy like 5-fluorouracil in MMR-deficient tumors. This personalized approach not only optimizes patient care but also enhances overall survival rates, especially for those diagnosed with stage II disease, underscoring the importance of genetic insights in improving surgical outcomes in LS-associated CRC.

The variability in MSI and IHC testing methods across studies introduces significant challenges in consistently interpreting pooled results. Different methodologies, such as single-marker versus multi-marker MSI panels and varied IHC protocols, can affect the detection rates of Lynch syndrome, especially for subtler MSH6 and PMS2 mutations known for their heterogeneous expression. This heterogeneity underscores the need for a focused discussion on how different testing strategies might influence study outcomes, guiding towards standardized genetic screening practices to enhance detection accuracy in younger CRC patients. Moreover, the lack of similar studies on LS mutations in the past 15 years could be attributed to the complexity and cost associated with conducting large-scale screening. Also, advances in genetic testing technology and shifts in research focus towards more targeted, personalized medicine approaches could also have redirected resources.

The decision to exclude studies focusing on individuals over 60 from this review is grounded in our aim to specifically investigate Lynch syndrome in younger populations, where early detection and intervention can profoundly impact outcomes. This focus helps clarify the unique clinical presentations and genetic variations seen in younger patients, particularly as they pertain to MSH6 and PMS2, which might present differently or later in life. Addressing this specific age group enhances our understanding of early-onset LS and aids in developing targeted preventative strategies.

These findings suggest that early and targeted screening for Lynch syndrome using MSI and IHC tests, complemented by BRAF mutation analysis, is crucial for identifying at-risk colorectal cancer patients under 60. Tailoring surgical and therapeutic interventions based on specific MMR gene mutations are advised. It is also important to acknowledge the ineffectiveness of standard 5-fluorouracil chemotherapy in MMR-deficient tumors, advocating for personalized treatment strategies.

### 3.2. Limitations

A principal limitation arises from the heterogeneity of screening protocols—single-marker *BAT-26* in some studies versus pentaplex or even full five-marker MSI panels in others. This inconsistency may affect reported MSI prevalence and hamper cross-study comparisons. Additionally, the proportion of “red flag” MSI+ tumors that proceed to formal germline testing varied, reflecting real-world challenges such as patient refusal or loss to follow-up. Consequently, actual LS rates in these under-60 cohorts may be underestimated. Another limitation is the number of studies that exist on this topic, with a total of five studies included in the final analysis, as well as the relatively short follow-up in certain studies, restricting the ability to assess long-term outcomes such as 10-year survival or late recurrences. Finally, differences in health system infrastructure—such as universal screening mandates in some settings versus discretionary testing in others—could introduce selection biases affecting the generalizability of each study’s results.

## 4. Materials and Methods

### 4.1. Search Strategy and Eligibility Criteria

We conducted a comprehensive search in electronic databases—including PubMed, Scopus, and Web of Science—to identify studies investigating Lynch syndrome in patients with CRC under 60 years of age. The primary objective was to capture data on MMR deficiency testing methods (MSI or IHC), *BRAF* mutation status, germline mutation confirmation, and clinical outcomes such as staging, survival, or recurrence. Our search period spanned from inception to January 2025. No language restrictions were applied initially, though only English-language articles were ultimately included because translation resources were not available in all cases.

We used Medical Subject Headings (MeSH) and text words to build specific search strings encompassing “colorectal cancer”, “Lynch syndrome”, “hereditary nonpolyposis colorectal cancer”, “HNPCC”, “microsatellite instability”, “MSH2”, “MLH1”, “MSH6”, “PMS2”, “BRAF”, “mismatch repair deficiency”, and “young-onset” or “under 60.” Additional records were identified by handsearching reference lists of relevant articles and contacting experts in the field. We also explored conference proceedings and abstracts to ensure comprehensiveness.

The PubMed search string was “(“Colorectal Neoplasms”[Mesh] OR “colorectal cancer”) AND (“Lynch Syndrome II”[Mesh] OR “Hereditary Nonpolyposis Colorectal Neoplasms”[Mesh] OR “Lynch syndrome” OR “HNPCC”) AND (“DNA Mismatch Repair”[Mesh] OR MMR OR MLH1 OR MSH2 OR MSH6 OR PMS2) AND (“Microsatellite Instability”[Mesh] OR MSI) AND (“young onset” OR “under 60”) AND (“mutation” OR “gene mutation”)”.

The Scopus search string was “TITLE-ABS-KEY (“colorectal cancer” AND (“Lynch syndrome” OR “hereditary nonpolyposis colorectal cancer” OR “HNPCC”) AND (“DNA mismatch repair” OR MMR OR MLH1 OR MSH2 OR MSH6 OR PMS2) AND (“microsatellite instability” OR MSI) AND (“young onset” OR “under 60”) AND (“mutation” OR “gene mutation”))”.

The Web of Science search string was “TS = (“colorectal cancer” AND (“Lynch syndrome” OR “hereditary nonpolyposis colorectal cancer” OR “HNPCC”) AND (“DNA mismatch repair” OR MMR OR MLH1 OR MSH2 OR MSH6 OR PMS2) AND (“microsatellite instability” OR MSI) AND (“young onset” OR “under 60”) AND (“mutation” OR “gene mutation”))”.

Studies were deemed eligible if they (1) primarily examined CRC patients younger than 60; (2) performed either MSI, IHC, or both for MMR deficiency; (3) reported on subsequent germline testing or at least used *BRAF* mutation analysis to exclude sporadic MSI; (4) included data on clinical characteristics or outcomes (e.g., stage at diagnosis, survival metrics, or response to therapy); and (5) were population-based or otherwise representative of a well-defined region or registry. We excluded case reports, purely descriptive studies lacking outcome data, or investigations focusing solely on older CRC populations. Initially, all citations identified were uploaded to a reference manager, and duplicates were removed. Two authors independently screened titles and abstracts, retaining only those fulfilling the inclusion criteria or requiring full-text review for clarification. Any disagreements were resolved by consensus or recourse to a third reviewer. Ultimately, five population-based studies met all the eligibility criteria for final inclusion.

### 4.2. Data Extraction and Quality Assessment

Two reviewers, each with experience in epidemiology and genetics, independently extracted data from the eligible full-text articles using a standardized form. The information collected encompassed basic study descriptors (author names, publication year, country, and design), patient demographics (total number, age range, and sex distribution), methods used to detect MMR deficiency (MSI testing panels, type of IHC performed, *BRAF* mutation protocols), and the proportion of tumors identified as MSI+ or IHC-deficient. We also recorded how many MSI+ or MMR-deficient tumors underwent further stratification by *BRAF* or *MLH1* hypermethylation to exclude likely sporadic MSI. Where available, we extracted the number of patients who proceeded to germline MMR gene testing, the distribution of pathogenic mutations found, and the correlated clinical outcomes such as tumor stage, survival rate, recurrence, and response to chemotherapy.

To evaluate the methodological rigor of the included studies, we adapted standard quality appraisal tools for observational designs, focusing on representativeness, sample selection, completeness of follow-up, and clarity of reporting. For population-based designs, we verified that each study adequately captured all relevant CRC cases under 60 within the chosen geographic region or registry timeframe. We also checked how thoroughly each study validated MSI or IHC results (e.g., confirmatory tests, second pathologist opinion, or double scoring) and whether potential confounders, such as family history or prior screenings, were documented. Risk of bias was rated as low, moderate, or high, with final determinations made by consensus. The overall quality of evidence was deemed sufficient to justify comparative interpretation, though we note that certain differences in testing protocols (e.g., single-marker *BAT-26* vs. multi-marker panels) might introduce variability in reported rates of MMR deficiency and subsequent outcomes.

### 4.3. Data Synthesis and Analysis

Given the heterogeneity across studies in both design and outcomes measured, we adopted a descriptive synthesis approach rather than a formal meta-analysis. We first summarized the demographic and methodological characteristics to highlight potential sources of variation (e.g., differences in MMR testing, *BRAF* screening, or inclusion criteria). Next, we tabulated the proportion of CRCs testing MSI+ or showing IHC loss and further delineated how many of those were classified as sporadic due to *BRAF* mutations or *MLH1* hypermethylation. We then documented how many patients underwent germline testing and how many had confirmed pathogenic mutations. This portion of the synthesis enabled us to approximate the yield of LS diagnoses for CRC patients under 60, factoring in the differences in each study’s approach.

Where outcome data were available, we grouped patients by gene mutated (*MLH1*, *MSH2*, *MSH6*, *PMS2*) and extracted survival rates, stage at diagnosis, recurrence data, or any reported prognostic hazard ratios. When absolute numeric outcome measures were lacking, we summarized the directionality of findings and relevant descriptive statistics. We further explored whether subgroups with *MSH2* or *MLH1* mutations differed from *MSH6* or *PMS2* carriers in terms of clinical presentation or therapy response. Whenever possible, we aligned definitions of end points (e.g., disease-free survival vs. recurrence-free survival) to facilitate comparisons. Because only a minority of studies provided explicit hazard ratios or long-term outcomes, we limited our interpretation to broad trends in LS-related survival and disease progression.

**Figure 2:**
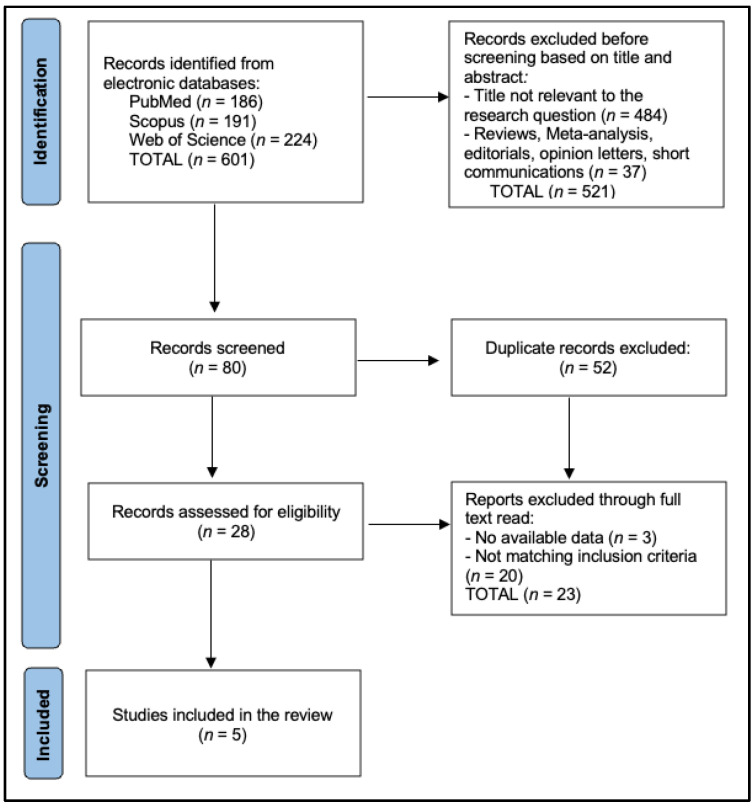
PRISMA flowchart.

In the present review, we provide aggregated or range-based data rather than attempting a pooled analysis. Heterogeneity across the studies in design, follow-up duration, and exact outcome definitions made meta-analysis impractical. Instead, we present central tendencies for the proportion of MSI+ tumors and LS diagnoses in under-60 CRC populations, acknowledging potential overlap or variation in measurement methods. We discuss any meaningful relationships identified between MMR mutation subtypes and outcomes but avoid overstating conclusions given the limited sample sizes for certain subgroups (particularly *MSH6* and *PMS2*). Where feasible, we note *p*-values and effect sizes from the original works to convey the statistical significance of observed trends. Interpretations within the subsequent results and discussion prioritize clarity, clinical relevance, and consistency with the underlying data. The study followed the PRISMA protocol, as presented in Figure 2.

## 5. Conclusions

Among CRC patients under 60, universal or near-universal MMR deficiency screening reliably identifies a clinically significant proportion of Lynch syndrome. Despite methodological variations, all five population-based investigations consistently report an improved prognosis for MMR-deficient patients, suggesting that earlier detection and more individualized adjuvant regimens could optimize outcomes. Distinctions in mutation patterns, notably among *MSH2*, *MLH1*, *MSH6*, and *PMS2*, underline the complexity of employing IHC alone, especially if heterogeneous staining is overlooked. Going forward, standardized testing protocols and collaborative registries are key to clarifying how best to integrate genetic findings, refine therapeutic decisions, and implement prophylactic measures for young patients with hereditary risk. Nevertheless, several study limitations such as the small number of studies included, the variability in diagnostic methodologies, and potential selection biases should be taken into account when interpreting these findings.

## Figures and Tables

**Table 1:** Study characteristics and MMR testing approaches.

Study and Author	Country/Region	Study Period	Design	Sample Size (*n*)	Age Range	MMR Testing Method
Schofield et al. [21]	Australia (W. Australia)	2000–2006	Population-based	1344	<60 years	MSI (*BAT-26*), BRAF, IHC (MLH1/PMS2/MSH2/MSH6)
Buchanan et al. [22]	Australia (Victoria)	1997–2009	Two prospective cohorts	1639 (total)	<60 primarily	MSI multi-marker, BRAF, IHC, germline
Schofield et al. [23]	Australia (W. Australia)	2009–2010	State-wide program	270	<60 years	MSI (pentaplex) + BRAF, IHC
Ward et al. [24]	Australia (Sydney)	1994–2004	Prospective single site	786 individuals (836 tumors)	<60 subset included	MSI multi-marker, IHC (MLH1/MSH2)
Watson et al. [25]	Australia (W. Australia)	2000–2004	Population-based	1003	<60 years	MSI (BAT-26), BRAF, IHC (all 4 MMR)

**Table 2:** Proportion of MSI and confirmed germline mutations in patients under 60.

Study	Total CRC < 60 (*n*)	MSI+ (%)	BRAF+ Among MSI+	Germline Testing	Mutation Yield
Schofield et al. [21]	1344	7.8	7/105 (6.7%)	98 “red flag” → 35 tested	11 newly discovered + 25 known = 36 total
Buchanan et al. [22]	ACCFR: 813; MCCS: 826	11.1 (ACCFR), 12.5 (MCCS)	NA	MMR-def tumors → germline	ACCFR: 5.2% carriers, MCCS: 0.8% carriers
Schofield et al. [23]	270	70/270 (26)	25/70 (35.7%)	45 “red flag” → 31 tested	15 carriers (48% tested)
Ward et al. [24]	786 individuals	115/836 (13.8)	Not stated	108 with loss on IHC → subset tested	∼0.8% for confirmed LS
Watson et al. [25]	1003	7.5	6/75 (8%)	69 MSI+/BRAF− → germline	18 known carriers, 3 newly ID’d (≥2.1%)

Mutation yield refers to germline mutation confirmation.

**Table 3:** Distribution of MMR gene mutations and patterns of protein loss.

Study	Major Genes Screened	Most Common Mutation(s)	IHC Loss Pattern	Comments
Schofield et al. [21]	*MLH1*, *MSH2*, *MSH6*, *PMS2*	*MSH2* and *MLH1* predominantly	Often dual loss: MLH1/PMS2 or MSH2/MSH6	Some cases of solitary PMS2 or MSH6 loss were noted.
Buchanan et al. [22]	*MLH1, MSH2*, *MSH6*, *PMS2*	*MLH1* or *MSH2* in the majority	Standard patterns, plus occasional heterogeneity	MSH6 and PMS2 carriers were a smaller fraction.
Schofield et al. [23]	*MLH1*, *MSH2*, *MSH6*, *PMS2*	15 carriers found: distribution not fully specified	Distinct patterns of loss consistent with germline	8/15 had single-protein losses (PMS2 or MSH6).
Ward et al. [24]	*MLH1*, *MSH2* primarily	*MLH1, MSH2* only	Rare solitary MSH6 or PMS2 not assessed widely	Study focused primarily on MLH1/MSH2 IHC.
Watson et al. [25]	*MLH1*, *PMS2*, *MSH2*, *MSH6*	18 known carriers, mostly *MSH2, MLH1*	51% had MLH1/PMS2 loss, 25% MSH2/MSH6 loss; 12% heterogeneous	Heterogeneous staining complicated interpretation.

**Table 4:** Clinical outcomes and survival in MMR-deficient vs. MMR-proficient patients under 60.

Study	MMR-Def Patients	Stage Distribution	Survival/Prognosis	Notable Findings
Schofield et al. [21]	7.8% MSI+ <60 years	More often Stage II or III but with fewer Stage IV	Improved overall survival among MMR-def. Some minimal 5-FU benefit reported.	Early detection critical; 36 total carriers identified.
Buchanan et al. [22]	11.1% (ACCFR) and 12.5% (MCCS) MMR-def	Stage II overrepresented among MMR-def	MMR-def tumors had better 5-year survival, especially if node-negative.	Lynch patients often had synchronous/metachronous disease.
Schofield et al. [23]	26% MSI+ (selective cohort)	Variable stage, but most “red flags” were earlier	MMR-def had a low mortality rate over short follow-up.	Subset analysis suggests no added benefit from 5-FU chemo.
Ward et al. [24]	13.8% MSI+ <60 subset	MMR-def frequently high grade but fewer metastases	MMR-def was an independent good prognostic factor (*p* < 0.05).	Adjuvant therapy utility questioned in stage II MMR-def.
Watson et al. [25]	7.5% MSI+ in <60 years	Predominantly right-sided, often Stage II	LS carriers had improved survival vs. sporadic MSI or MSS patients.	Heterogeneous IHC patterns complicated staging correlation.

## Data Availability

Not applicable.
